# Risk Factors of Suicidal Behaviour in old age

**DOI:** 10.1192/j.eurpsy.2022.112

**Published:** 2022-09-01

**Authors:** M. Lozupone, A. Mollica, G. Berardino, A. Bellomo, F. Panza

**Affiliations:** 1 University of Bari Aldo Moro, Neurodegenerative Disease Unit, Bari, Italy; 2 Psychiatric Unit, University of Foggia, Department Of Clinical And Experimental Medicine, Foggia, Italy; 3 University of Foggia, Department Of Clinical And Experimental Medicine, Psychiatric Unit, Foggia, Italy

**Keywords:** epigenetics, older age, Late-life depression, suicidal behaviour

## Abstract

Several observational studies investigated risk factors for suicide attempts/completed suicides in older age with contrasting evidence from ongoing population-based research. The risk factors most associated to suicide attempts than other variables were: depressive disorders, methods employed to self-harm (particularly poisoning), and psychotropic drug utilization followed by psychological factors and disability. Moreover, male sex, violent methods to self-harm, any psychiatric disorder (depression, anxiety and bipolar disorders), a poor medical condition, stressors/bereavement, and living alone appeared to be more significant for predicting completed suicides in late life. There is growing evidence of a role of environmental exposures in the pathogenesis and epigenetics of suicidal behavior in older age. Little is known about the possible relationship between suicidal ideation in older age and its biopsychosocial predictors, although psychiatric disorders (among which late-life depression, LLD), play a fundamental role. LLD, distinguished as late-onset depression (LOD) and early-onset depression (EOD). Suicidal ideators accounted for 2.32% of subjects, were female, smokers and obese affected by multimorbidity. After adjusting for age, gender, education and social dysfunction, suicidal ideation was associated to LLD (EOD>LOD:OR:21.71, 95% CI:9.22-51.14). In the full random forest model, asthma was the most important contributor to suicidal ideation. Among biomarkers, interleukin (IL)-6 followed by tumor necrosis factor (TNF)-a, Apolipoprotein E e4 allele-carriers, C-reactive protein contributed most to suicidal ideation. Although EOD is a strong determinant of suicidal ideation, other non-psychiatric factors, i.e., serum inflammation biomarkers, APOE e4 allele, and multimorbidity, should be taken into account when evaluating a suicidal ideation phenotype in older age.

**Disclosure:**

No significant relationships.

